# A propensity score matching study on survival benefits of radiotherapy in patients with inoperable hepatocellular carcinoma

**DOI:** 10.1038/s41598-023-34135-6

**Published:** 2023-04-27

**Authors:** Hao Zeng, Ke Su, Xiaojing Chen, Xueting Li, Lianbin Wen, Yanqiong Song, Lan Chen, Han Li, Lu Guo, Yunwei Han

**Affiliations:** 1 Department of Oncology, The Affiliated Hospital of Southwest Medical University, No.25 Taiping Street, Luzhou, Sichuan Province China; 2 Department of Oncology, 363 Hospital, Chengdu, China; 3 Department of Geriatric Cardiology, Sichuan Academy of Medical Sciences & Sichuan Provincial People’s Hospital, Chengdu, China; 4 Department of Radiotherapy, School of Medicine, Sichuan Cancer Hospital & Institute, Sichuan Cancer Center, University of Electronic Science and Technology of China, Chengdu, China; 5 Department of Oncology and Hematology, The Affiliated Traditional Chinese Medicine Hospital of Southwest Medical University, Luzhou, China; 6 Department of Ophthalmology, The Affiliated Hospital of Southwest Medical University, Luzhou, China

**Keywords:** Cancer, Cancer therapy, Radiotherapy

## Abstract

With the advancements in radiotherapy (RT) in recent years, several studies have shown that RT can significantly prolong the survival of patients with hepatocellular carcinoma (HCC). As a noninvasive treatment option, the application of RT for the treatment of HCC is garnering increasing attention. In this retrospective study, we included data from 13,878 patients with HCC from the Surveillance, Epidemiology, and End Results (SEER) database between 2000 and 2019 and 325 patients with HCC treated in three tertiary hospitals in China between 2015 and 2021. Patient data were divided into RT and non-RT groups based on whether the patients underwent RT. Propensity score matching analysis was performed to minimize the deviation between the RT and non-RT groups, and the Kaplan–Meier method, Cox proportional hazard model, and nomogram were used to assess the efficacy of undergoing RT. The median overall survival (mOS) of the RT group was significantly longer compared with that of the non-RT group for the SEER data (16 months versus 9 months, p < 0.01). Similarly, the survival benefit was more significant in the RT group than in the non-RT group at our hospitals (34.1 months versus 15.4 months, p < 0.01). Furthermore, multivariate Cox analysis revealed that factors, including tumor (T) stage, patient age, tumor grade, serum AFP level, and chemotherapy, also affected patient survival. Moreover, these factors were also used to construct a nomogram. Subgroup analysis of these factors showed that RT was effective in prolonging patient survival in different populations. RT significantly improves the survival time of patients with inoperable HCC, thereby providing a basis for selecting HCC patients who can benefit from RT.

## Introduction

Hepatocellular carcinoma (HCC) is the sixth most common malignancy and the second leading cause of tumor-related mortality^1^. Surgical treatment, including hepatectomy and liver transplantation, remains the optimal treatment option for patients with HCC. Many large retrospective studies have shown that HCC patients exhibit a 5-year survival rate of > 50% following partial hepatectomy^2–4^. However, owing to the scarcity of liver grafts, the location, size, and number of tumors as well as the impairment of the liver function of the patients, a significant proportion of patients are ineligible for surgical treatment; therefore more effective nonsurgical treatments should be selected for them^5^.

The main nonsurgical treatment modalities for patients with HCC encompass systemic therapies and locoregional therapies. The National Comprehensive Cancer Network (NCCN) guidelines have listed the combination of atezolizumab and bevacizumab as a preferred chemotherapeutic regimen for advanced or unresectable HCC^6^. Locoregional therapies are broadly categorized into radiofrequency ablation (RFA), transarterial chemoembolization (TACE), and radiotherapy (RT). The guidelines from the European Association for the Study of the Liver and the American Association for the Study of Liver Diseases have accepted RFA as a first choice of treatment for patients with early-stage HCC who are ineligible for surgical treatment^7–9^. In carefully selected patients with HCC, RFA can even achieve radical results that are comparable to surgery^10,11^. TACE is another option for locoregional treatments of patients with HCC. Chung-Mau et al. reported that actuarial survival was significantly better in the TACE group (1 year, 57%; 3 years, 26%) than in the supportive care group (1 year, 32%; 3 years, 3%; p = 0.002)^12^. However, as invasive treatment modalities, RFA and TACE have stringent requirements for the liver functional status of patients and greatly affect the quality of life of patients after the procedure. Therefore, RT, as a noninvasive locoregional therapy for HCC, holds unique advantages over RFA and TACE.

RT functions by disrupting the double-stranded structure of DNA and also by activating CD8 T cells via the release of tumor-associated antigens, which results in immunogenic cell death^13,14^. RT options for HCC include stereotactic body radiotherapy (SBRT), intensity-modulated radiation therapy (IMRT), and gamma knife radiosurgery (GKR)^15^. Previous studies suggest that the survival benefit of SBRT for patients with early to mid-stage HCC is comparable to that of surgical resection or RFA^16–18^. Furthermore, Lu et al. first reported that the combination of TACE and GKR was well tolerated and yielded overall survival (OS) benefits in patients with HCC having portal vein tumor thrombosis (PVTT)^15^. In our previous study, Su et al. demonstrated that GKR provided better OS compared with that TACE in patients with HCC having PVTT (17.2 versus 8.0 months, p < 0.001)^19^. RT is an evolving technique for HCC and demands more attention.

In this study, survival data from the Surveillance, Epidemiology, and End Results (SEER) database (National Cancer Institute [NCI], Bethesda, MD, USA) and patients with inoperable HCC at three Chinese tertiary hospitals were incorporated. Survival data were compared between the group that underwent RT and the group that did not undergo RT, and factors affecting OS in these patients were evaluated. Through this data, we aim to provide additional survival estimates and prognostic insights for the efficacy of RT in patients with inoperable HCC, generate testable hypotheses for more precise clinical trials, and consequently identify which patients with HCC will most likely be benefitted from RT.

## Materials and methods

### Data source and study cohort

Herein, we obtained specific clinicopathological data and prognostic outcomes of patients with HCC from 2000 to 2019 using the program SEER*Stat (Version 8.4.0, NCI, Bethesda, MD, USA). Cases of HCC diagnosed from 2000 to 2019 were obtained from the SEER database (Incidence-SEER Research Plus Data, 17 Registries, Nov 2021 Sub [2000–2019]).

The inclusion criteria for SEER data were as follows: (1) International Classification of Disease for Oncology, Third Edition (ICD-O-3) histology codes 8170/3 and 8172/3–8175/5, with the liver site code C22.0. Fibrolamellar histology (8171/3) was excluded owing to its significant differences from conventional HCC^20^; (2) no cancer in nearby lymph nodes (N0) and no metastasis (M0); and (3) age of patients > 18 years. The study exclusion criteria were as follows: (1) age > 85 years; (2) patients who had undergone surgery; (3) incomplete survival data; (4) race unknown; and (5) no information on whether the patient had undergone RT. Subsequently, we divided the patient data into RT and non-RT groups.

Data were also collected from patients with inoperable HCC who underwent RT and other treatments between June 2015 and July 2021 at three tertiary care hospitals in China. These patients were divided into RT and non-RT groups. Patients in the RT group were primarily treated with GKR, whereas those in the non-RT group mainly underwent treatment modalities such as included TACE, RFA, and systemic therapies.

The study inclusion criteria for these patients admitted at the hospitals were as follows: (1) age ≥ 18 years, (2) HCC diagnosed by histology or cytology, (3) HCC evaluated as inoperable or patient unwilling to undergo surgery, (4) Child–Pugh class A/B, and (5) Eastern Cooperative Oncology Group Performance Status score of 0–2. Furthermore, the patient exclusion criteria were as follows: (1) tumor cells found in one regional lymph node (N1) or cancer metastasized to one other part of the body (M1), (2) presence of diffuse disease or more than five tumor nodes, (3) those in whom GKR or other treatments could not be adequately performed, and (4) those with incomplete clinical information. The study design was approved by the Ethics Committee of the Affiliated Hospital of Southwest Medical University (approval number KY2020254) and adhered to the standards of the Declaration of Helsinki. Written informed consent was waived because of the retrospective nature of the study.

### Variables collected

Information on the following parameters was collected from the SEER database: (1) patient age at the time of diagnosis, (2) gender (Female/Male), (3) ethnicity (White/Black/Other), (4) marital status (Married/Single/Divorced/Widowed/Other-Unknown), (5) tumor (T) stage (T1/T2/T3/T4), (6) tumor differentiation grade (I-II/III-IV/Unknown), (7) serum alpha-fetoprotein (AFP) level (Elevated/Normal/Unknown), (8) received chemotherapy (Yes/No), (9) received RT (Yes/No), and (10) OS in months (the duration from diagnosis to death from any cause). The SEER database did not provide any information on the chemotherapy regimen or the quality of life of the patients. The closing date used for follow-up data was December 31, 2019.

Besides patient age at diagnosis, gender, and serum AFP level, we also collected information on other parameters including Child–Pugh score, tumor size, tumor stage, PVTT type, presence of hepatitis B and/or hepatitis C viruses, and alcohol consumption by patients with HCC at the three Chinese tertiary hospitals.

### Treatment protocol

#### GKR

The treatment protocol for GKR has been described in our previous study^19^. Briefly, the gross tumor volume (GTV) encompassing the primary liver tumor was delineated using imaging technology. A 5–10-mm boundary around the GTV was defined as the planned target volume. The median radiation dose received by the patients was 42 Gy (range: 39–42 Gy), and the median isodose line was 50% (range: 50–60%).

### Statistical analysis

The study subjects were matched by propensity score matching (PSM) to their nearest neighbor in a 1:1 ratio without replacement. This strategy reduced the potential bias associated with selecting specific patients to undergo RT by comparing survival outcomes between the matched groups of patients with or without RT. The validity of PSM was assessed by comparing the RT and non-RT groups for each selected variable before and after PSM implementation. All variables were treated as categorical and analyzed by chi-squared (χ^2^) tests before PSM and by McNemar’s test after PSM.

The Kaplan–Meier (KM) method was followed to estimate OS. Differences between the median OS (mOS) of the RT subgroups were evaluated by performing the log-rank test^21,22^. Hazard ratios for death were estimated using a Cox proportional hazard model with predictor variables^23^. A nomogram was constructed on the basis of independent risk factors identified by performing multivariate Cox analysis^24,25^. A two-sided p-value < 0.05 was considered statistically significant.

## Results

### Selection of the study cohort and PSM from the SEER database

We extracted data from 13,878 patients with HCC without metastasis and who had not undergone surgery at the time of diagnosis from the SEER database. Among these patients, 5025 (36.2%) were below 60 years of age, 5190 (37.4%) were between 60 and 69 years of age, and 3663 (26.4%) were above 70 years but below 85 years of age. The male-to-female ratio was 3.5:1. Other parameters were as follows: A total of 9858 (71%) patients were of white ethnicity, 6659 (48.0%) were married, and 6160 (44.4%) showed T1-stage tumors. Tumor grade was unknown in 10,473 (75.5%) patients and was grade I-II in 2689 (19.4%) patients. Most patients (11,888 [85.7%]) showed only one tumor at the primary site. A total of 8617 (62.1%) patients showed increased serum AFP levels. About half of the patients (7440 [53.6%]) had undergone chemotherapy. The RT group consisted of 1381 (10.0%) patients, whereas the non-RT group included 12,497 (90.0%) patients (Table 1).

**Table 1:** Baseline patient characteristics before and after propensity score matching (PSM).

Factors	Pre-PSM				RT (n = 1381)	Post-PSM			
	Non-RT (n = 12,497)	Overall (n = 13,878)	Comparison			Comparison		Non-RT (n = 1381)	Overall (n = 2762)
			*X*^2^	P		*X*^2^	P		
Age at diagnosis (n, %)									
< 60 years	4613 (36.9)	5025 (36.2)	29.99	< 0.01	412 (29.8)	0.20	0.91	409 (29.6)	821 (29.7)
≥ 60, < 70 years	4646 (37.2)	5190 (37.4)			544 (39.4)			555 (40.2)	1099 (39.8)
≥ 70, < 85 years	3238 (25.9)	3663 (26.4)			425 (30.8)			417 (30.2)	842 (30.5)
Gender (n, %)									
Female	2819 (22.6)	3082 (22.2)	8.68	< 0.01	263 (19.0)	0.01	0.92	260 (18.8)	523 (18.9)
Male	9678 (77.4)	10,796 (77.8)			1118 (81.0)			1121 (81.2)	2239 (81.1)
Ethnicity (n, %)									
White	8833 (70.7)	9858 (71.0)	7.72	0.02	1025 (74.2)	0.09	0.96	1029 (74.5)	2054 (74.4)
Black	1718 (13.7)	1881 (13.6)			163 (11.8)			158 (11.4)	321 (11.6)
Others	1946 (15.6)	2139 (15.4)			193 (14.0)			194 (14.0)	387 (14.0)
Marital status (n, %)									
Married	5859 (46.9)	6659 (48.0)	70.30	< 0.01	800 (57.9)	0.92	0.92	810 (58.7)	1610 (58.3)
Single	2871 (23.0)	3081 (22.2)			210 (15.2)			217 (15.7)	427 (15.5)
Divorced	1670 (13.4)	1841 (13.3)			171 (12.4)			156 (11.3)	327 (11.8)
Widowed	1059 (8.5)	1159 (8.4)			100 (7.2)			97 (7.0)	197 (7.1)
Others/Unknown	1038 (8.3)	1138 (8.2)			100 (7.2)			101 (7.3)	201 (7.3)
T Stage (n, %)									
T1	5666 (45.3)	6160 (44.4)	56.50	< 0.01	494 (35.8)	2.11	0.55	489 (35.4)	983 (35.6)
T2	3028 (24.2)	3381 (24.4)			353 (25.6)			354 (25.6)	707 (25.6)
T3	3454 (27.6)	3950 (28.5)			496 (35.9)			511 (37.0)	1007 (36.5)
T4	349 (2.8)	387 (2.8)			38 (2.8)			27 (2.0)	65 (2.4)
Grade (n, %)									
Grade I-II	2356 (18.9)	2689 (19.4)	24.02	< 0.01	333 (24.1)	1.06	0.59	320 (23.2)	653 (23.6)
Grade III-IV	638 (5.1)	716 (5.2)			78 (5.6)			69 (5.0)	147 (5.3)
Unknown	9503 (76.0)	10,473 (75.5)			970 (70.2)			992 (71.8)	1962 (71.0)
Serum AFP (n, %)									
Elevated	7721 (61.8)	8617 (62.1)	5.78	0.06	896 (64.9)	0.73	0.70	917 (66.4)	1813 (65.6)
Normal	2649 (21.2)	2928 (21.1)			279 (20.2)			269 (19.5)	548 (19.8)
Unknown	2127 (17.0)	2333 (16.8)			206 (14.9)			195 (14.1)	401 (14.5)
Chemotherapy (n, %)									
Yes	6821 (54.6)	7440 (53.6)	47.23	< 0.01	619 (44.8)	0.01	0.94	622 (45.0)	1241 (44.9)
No	5676 (45.4)	6438 (46.4)			762 (55.2)			759 (55.0)	1521 (55.1)

*RT* radiotherapy, *PSM* propensity score matching, *AFP* alpha-fetoprotein.

**Figure 1:**
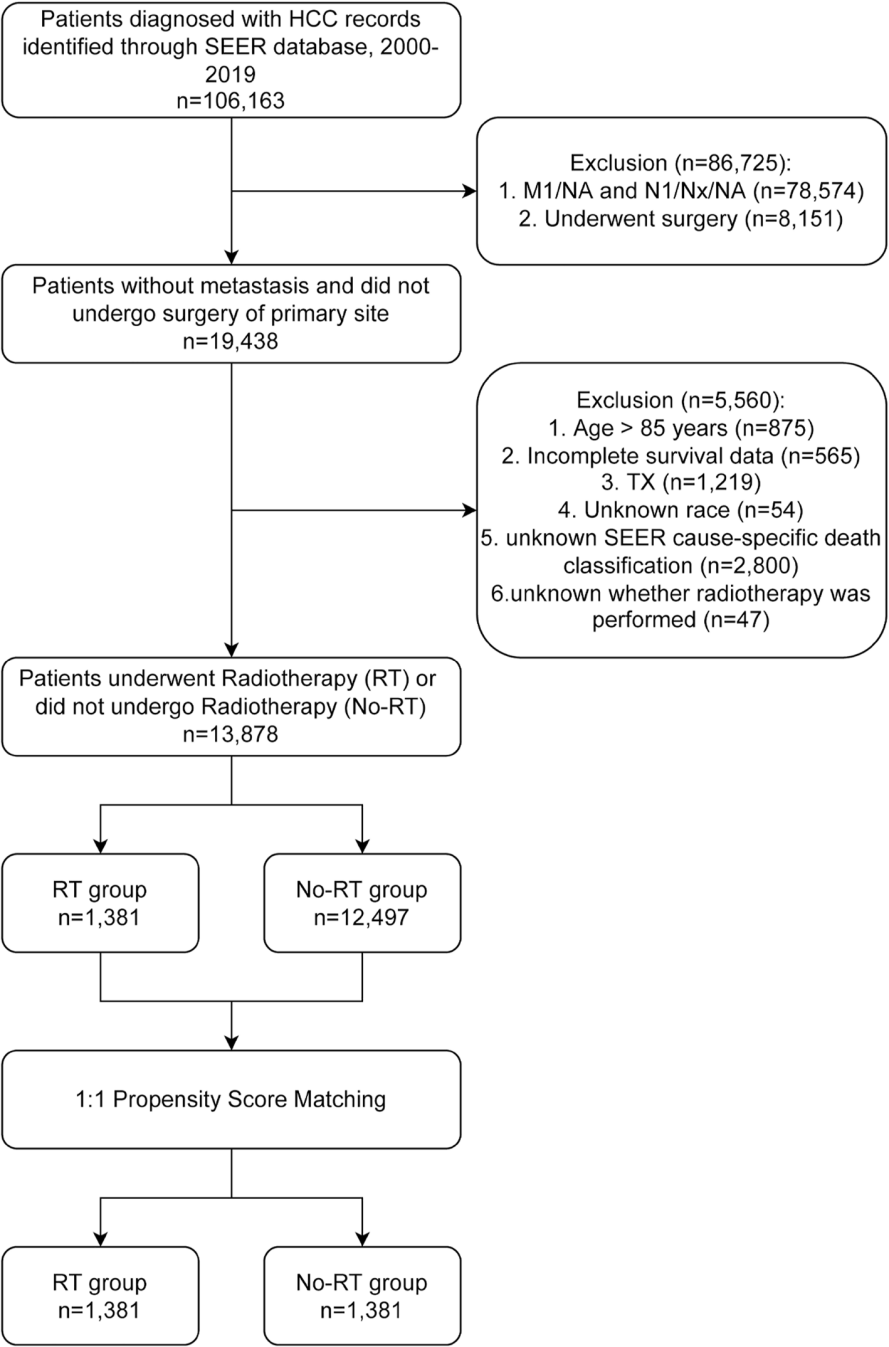
Flow chart depicting the patient selection process. *RT* radiotherapy.

The post-PSM cohort consisted of 2762 patients, and differences in all predictor variables were eliminated (Table 1, Fig. 1). Thus, PSM may effectively minimize the effects of potential confounding factors.

### Survival outcomes after PSM from the SEER database

**Figure 2:**
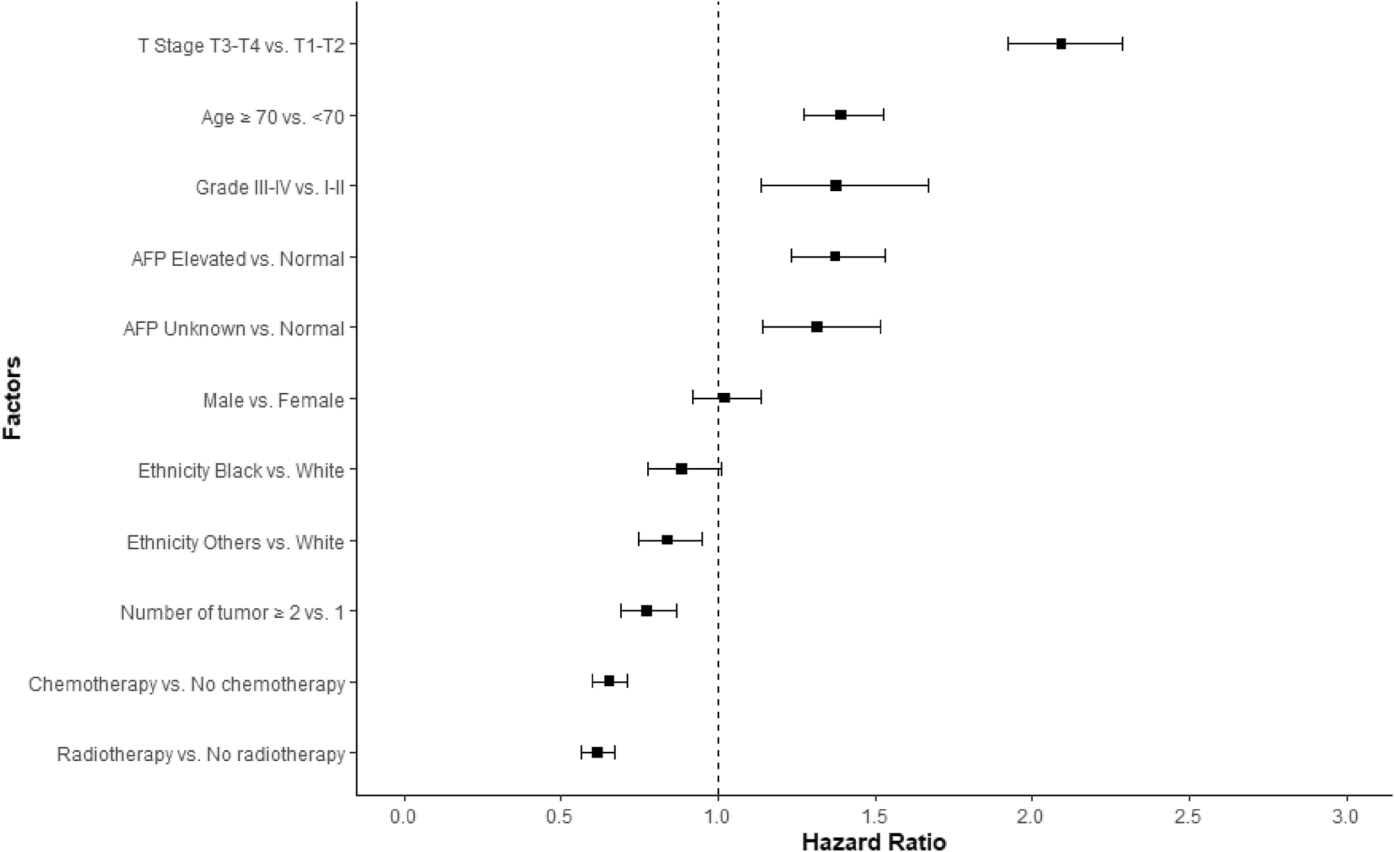
Cox proportional hazard ratios with 95% confidence intervals for data from the SEER database. *AFP* alpha-fetoprotein.

**Figure 3:**
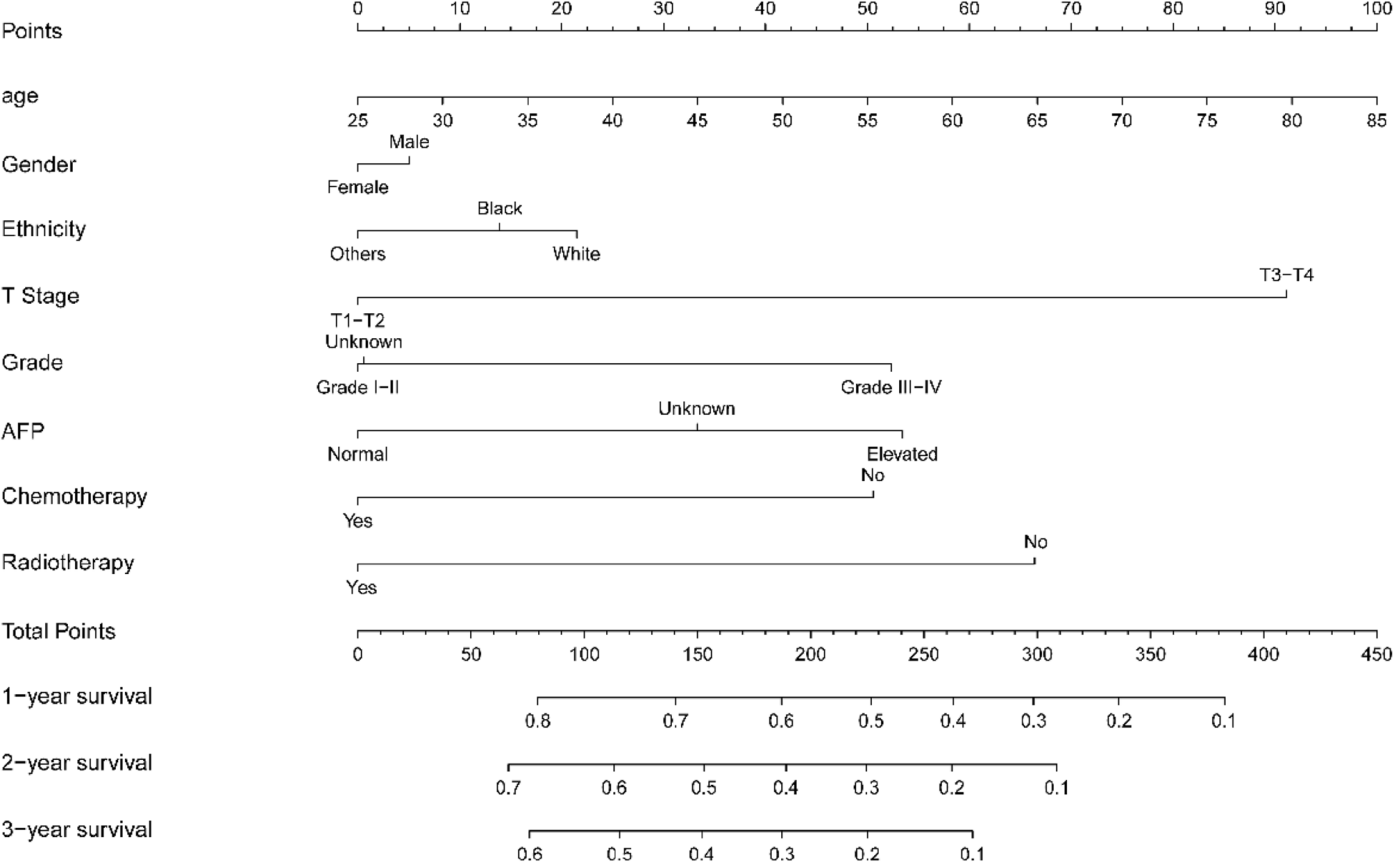
Nomogram constructed based on independent risk factors, as determined by multifactor Cox analysis. *AFP* alpha-fetoprotein.

Following PSM, the mOS of the RT group (16 months, 95% confidence interval [CI] 15–18) was significantly longer than that of the non-RT group (9 months, 95% CI 7–10, p < 0.05). Furthermore, in the multivariate Cox regression model, the following six factors were identified as major predictors of survival: (1) T stage, (2) patient age, (3) tumor grade, (4) serum AFP level, (5) chemotherapy, and (6) RT (Fig. 2). These factors subsequently contributed to nomogram construction, providing a graphical representation of the multivariate Cox model for survival (Fig. 3). In the validation set, we determined that the area under the curve values of predicted 1-, 2- and 3-year survival rates of the receiver operating characteristic curve on the basis of the nomogram were 0.728, 0.709 and 0.709, respectively (Fig S1). Additionally, calibration curves in Fig S2 indicate the good prediction potential of this model.

**Figure 4:**
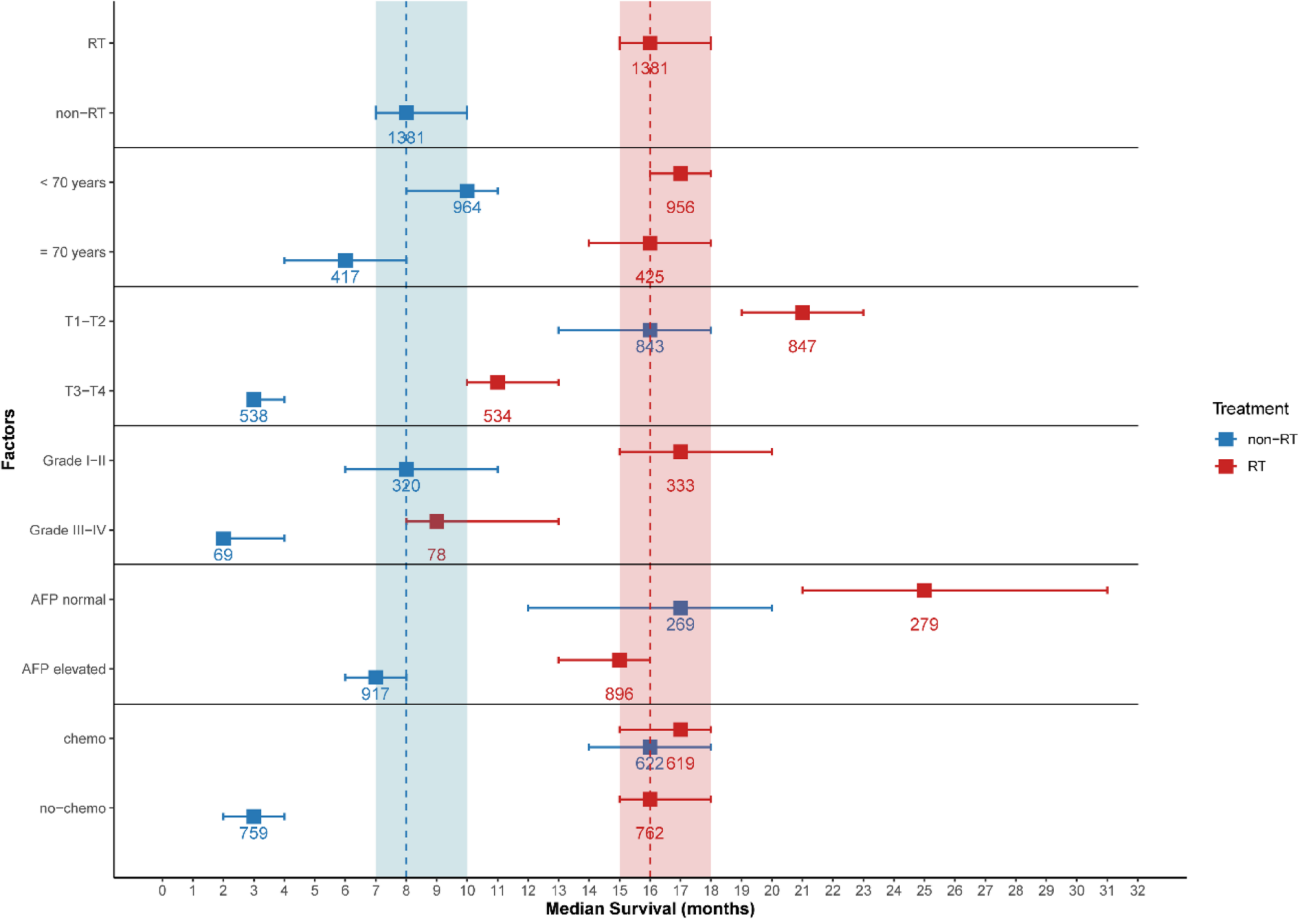
Median overall survival (months) ± 95% confidence intervals for patients in RT and non-RT. *RT* radiotherapy, *AFP* alpha-fetoprotein.

**Figure 5:**
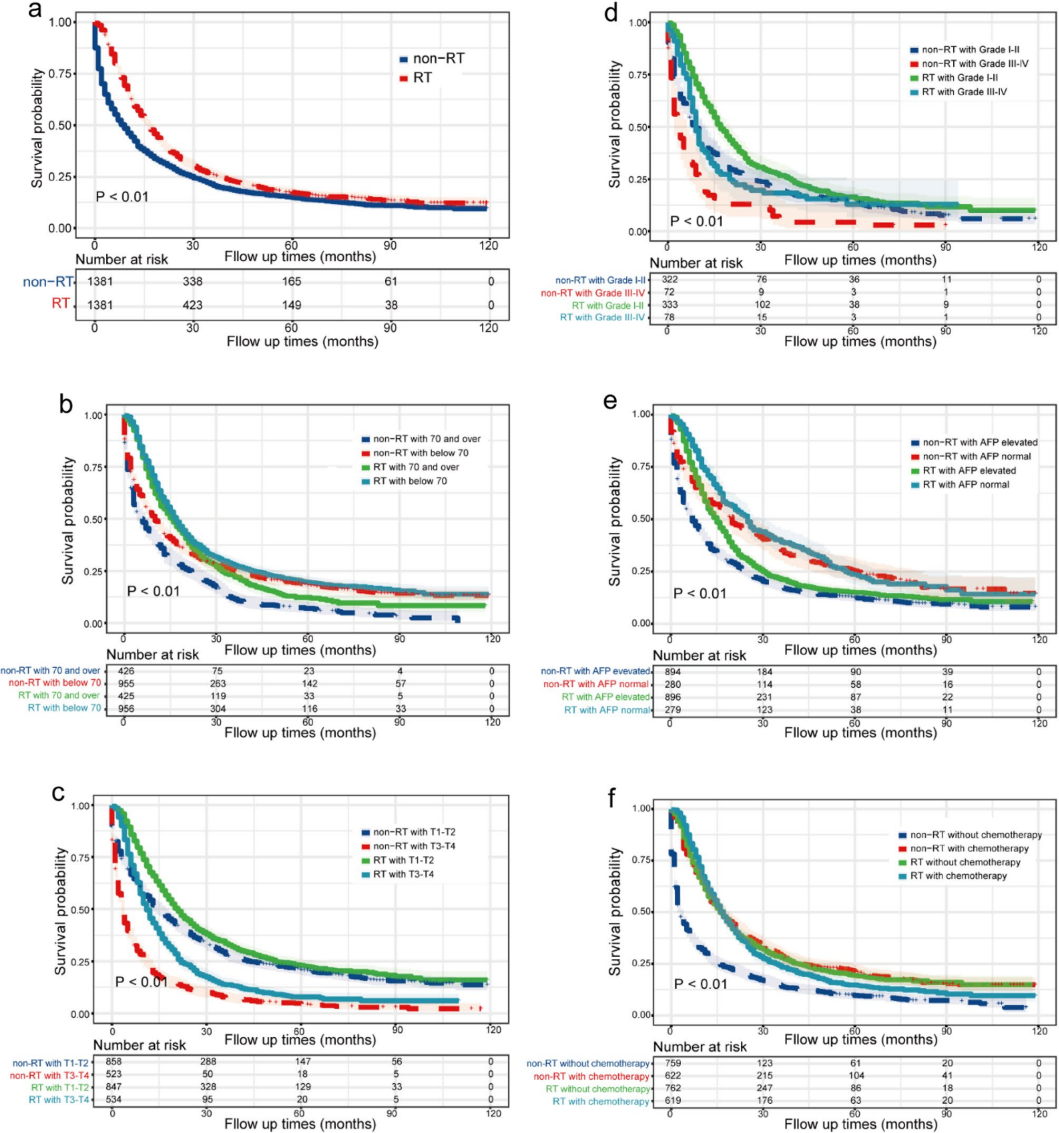
Kaplan–Meier overall survival (OS) estimates and 95% confidence intervals for patients in the RT and non-RT groups: (**a**) in the total sample and grouped by (**b**) age, (**c**) tumor (T) stage, (**d**) tumor differentiation grade, (**e**) serum AFP level, and (**f**) receipt of chemotherapy. *RT* radiotherapy, *AFP* alpha-fetoprotein.

To evaluate the effects of these factors between the RT and non-RT groups, we plotted the KM OS curve of different subgroups (Figs. 4, 5). RT effectively prolonged the survival time of patients younger and older than 70 years of age. The mOS of patients who underwent RT in the subgroup younger than 70 years was 17 months (95% CI 16–18), which was much longer than patients who did not undergo RT in the same age subgroup (6 months, 95% CI 4–8, p < 0.05) (Fig. 5b). In the T-stage subgroups, the longest mOS (21 months, 95% CI 19–23, p < 0.05) was observed in combination with RT in patients with the stage T1-T2 (Fig. 5c). Similarly, RT was effective in prolonging the mOS of patients in the well- and poorly-differentiated tumor grade subgroups (Fig. 5d). Patients who underwent RT and had normal serum AFP levels (25 months, 95% CI 21–31) showed much longer mOS than patients who did not undergo RT and had increased serum AFP levels (7 months, 95% CI 6–8, p < 0.05) (Fig. 5e). However, while analyzing chemotherapy combined with RT, no significant difference was observed between the mOS of those receiving one treatment modality alone (either RT or chemotherapy) or both modalities (RT alone: 16 months [95% CI 15–18], chemotherapy alone: 16 months [95% CI 14–18], and RT + chemotherapy: 17 months [95% CI 15–18]); however, mOS was much lower in patients who did not undergo either of the treatment modalities (3 months, 95% CI 2–4, p < 0.05) (Fig. 5f).

### Study cohort and survival outcomes from our hospitals

We screened and collected information on 325 patients with HCC from three tertiary hospitals in China, including 158 in the RT group and 167 in the non-RT group. A total of 151 of the 158 patients underwent GKR as their RT modality.

The number of patients enrolled in the RT and non-RT groups was 130 after PSM (Table 2).

**Table 2:** Baseline patient characteristics at three tertiary hospitals in China.

Factors	Pre-PSM					Post-PSM				
	RT (n = 158)	Non-RT (n = 167)	Overall (n = 325)	Comparison		RT (n = 130)	Non-RT (n = 130)	Overall (n = 260)	Comparison	
				*X*^2^	P				*X*^2^	P
Age at diagnosis (n, %)										
< 60 years	101 (60.5)	81 (51.3)	182 (56.0)	3.15	0.21	76 (58.5)	68 (52.3)	144 (55.4)	1.11	0.57
≥ 60, < 70 years	45 (26.9)	56 (35.4)	101 (31.1)			39 (30.0)	43 (33.1)	82 (31.5)		
≥ 70 years	21 (12.6)	21 (13.3)	42 (12.9)			15 (11.5)	19 (14.6)	34 (13.1)		
Gender (n, %)										
Female	20 (12.0)	22 (13.9)	42 (12.9)	0.13	0.72	13 (10.0)	19 (14.6)	32 (12.3)	0.89	0.35
Male	147 (88.0)	136 (86.1)	283 (87.1)			117 (90.0)	111 (85.4)	228 (87.7)		
BCLC (n, %)										
A	12 (7.2)	38 (24.1)	50 (15.4)	17.82	< 0.01	12 (9.2)	21 (16.2)	33 (12.7)	2.82	0.24
B	45 (26.9)	33 (20.9)	78 (24.0)			32 (24.6)	29 (22.3)	61 (23.5)		
C	110 (65.9)	87 (55.1)	197 (60.6)			86 (66.2)	80 (61.5)	166 (63.8)		
Child–Pugh stage (n, %)										
A	141 (84.4)	122 (77.2)	263 (80.9)	2.29	0.13	107 (82.3)	101 (77.7)	208 (80.0)	0.60	0.44
B	26 (15.6)	36 (22.8)	62 (19.1)			23 (17.7)	29 (22.3)	52 (20.0)		
Tumor diameter (cm, n, %)										
< 2	5 (3.0)	8 (5.1)	13 (4.0)	27.94	< 0.01	5 (3.8)	4 (3.1)	9 (3.5)	4.60	0.20
≥ 10	59 (35.3)	30 (19.0)	89 (27.4)			33 (25.4)	30 (23.1)	63 (24.2)		
≥ 2, < 5	36 (21.6)	75 (47.5)	111 (34.2)			36 (27.7)	52 (40.0)	88 (33.8)		
≥ 5, < 10	67 (40.1)	45 (28.5)	112 (34.5)			56 (43.1)	44 (33.8)	100 (38.5)		
Serum AFP (ng/ml, n, %)										
< 200	76 (45.5)	93 (58.9)	169 (52.0)	5.95	0.05	69 (53.1)	71 (54.6)	140 (53.8)	0.07	0.97
≥ 200, < 400	12 (7.2)	10 (6.3)	22 (6.8)			10 (7.7)	10 (7.7)	20 (7.7)		
≥ 400	79 (47.3)	55 (34.8)	134 (41.2)			51 (39.2)	49 (37.7)	100 (38.5)		
PVTT type (n, %)										
0	57 (34.1)	71 (44.9)	128 (39.4)	5.75	0.22	44 (33.8)	50 (38.5)	94 (36.2)	2.02	0.73
I	15 (9.0)	18 (11.4)	33 (10.2)			13 (10.0)	17 (13.1)	30 (11.5)		
II	43 (25.7)	32 (20.3)	75 (23.1)			32 (24.6)	30 (23.1)	62 (23.8)		
III	34 (20.4)	25 (15.8)	59 (18.2)			28 (21.5)	21 (16.2)	49 (18.8)		
IV	18 (10.8)	12 (7.6)	30 (9.2)			13 (10.0)	12 (9.2)	25 (9.6)		
HBV (n, %)										
Yes	103 (61.7)	107 (67.7)	210 (64.6)	1.05	0.31	91 (70.0)	86 (66.2)	177 (68.1)	0.28	0.59
No	64 (38.3)	51 (32.3)	115 (35.4)			39 (30.0)	44 (33.8)	83 (31.9)		
HCV (n, %)										
Yes	4 (2.4)	6 (3.8)	10 (3.1)	0.17	0.68	2 (1.5)	2 (1.5)	4 (1.5)	0.00	1.00
No	163 (97.6)	152 (96.2)	315 (96.9)			128 (98.5)	128 (98.5)	256 (98.5)		
Aclhole (n, %)										
Yes	61 (36.5)	73 (46.2)	134 (41.2)	2.75	0.10	55 (42.3)	54 (41.5)	109 (41.9)	0.00	1.00
No	106 (63.5)	85 (53.8)	191 (58.8)			75 (57.7)	76 (58.5)	151 (58.1)		
Radiotherapy modalities (n, %)										
GKR	151 (95.6)					125 (96.2)				
SBRT	3 (1.9)					2 (1.5)				
IMRT	4 (2.5)					3 (2.3)				

*RT* radiotherapy, *PSM* propensity score matching, *AFP* alpha fetoprotein, *PVTT* portal vein tumor thrombus, *HBV* hepatitis B virus, *HCV* hepatitis C virus, *GKR* gamma knife radiosurgery, *SBRT* stereotactic body radiotherapy, *IMRT* intensity-modulated radiation therapy.

Interestingly, the multivariate Cox results showed that most of the included factors did not affect the survival of patients with HCC (Fig S3). The survival analysis revealed that 57 (36.08%) patients in the RT group and 105 (62.87%) in the non-RT group died before PSM as of September 1, 2021.

**Figure 6:**
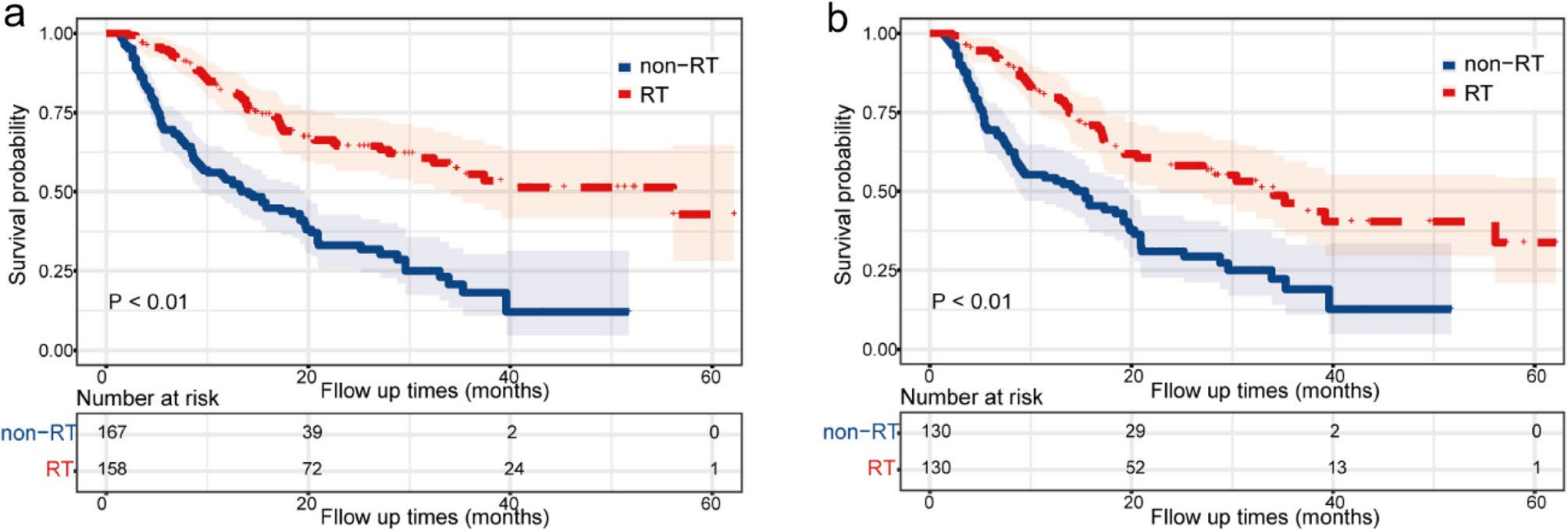
Kaplan–Meier overall survival (OS) estimates and 95% confidence intervals for patients in the RT and non-RT groups at three tertiary hospitals in China: (**a**) pre-PSM and (**b**) post-PSM. *PSM* propensity score matching, *RT* radiotherapy.

The mOS of the RT group versus that of the non-RT group was 56.1 months versus 13.4 months (p < 0.01) before PSM (Fig. 6a). After PSM, 54 (41.54%) patients in the RT group died compared with 81 (62.31%) patients in the non-RT group. The mOS of RT versus that of non-RT post-PSM was 34.1 months versus 15.4 months (p < 0.01) (Fig. 6b). Notably, the mOS of the GKR group was 39.2 months (Fig S4).

## Discussion

Here, we innovatively integrated data from the SEER database and patients at three Chinese tertiary hospitals to analyze the potential survival benefits of RT for patients with inoperable HCC. We showed that RT was more effective overall in prolonging patient survival owing to the information obtained from the SEER database and the patients at our hospitals. Furthermore, we identified several factors affecting OS, such as patient age, serum AFP level, tumor differentiation (i.e. grade), and T stage using the SEER database. Interestingly, most of these factors did not show statistically significant differences in patients at our Chinese hospitals, which might be attributed to the small sample size.

Notably, we are the first to amalgamate data from the SEER database with that of Chinese patients with HCC, providing a more comprehensive understanding of RT efficacy in inoperable patients with HCC and factors that affect patient outcomes. Based on the multivariate Cox regression analysis, nomogram construction, and KM survival analysis, we emphasized the positive effect of RT on patient prognosis across various subgroups, considering diverse factors affecting OS. Our findings suggest that RT, especially GKR, can serve as a dependable alternative for numerous patients with HCC who cannot undergo surgical intervention, thus laying the groundwork for future studies to explore and optimize the beneficial aspects of RT for nonsurgical patients with HCC.

A retrospective study performed by Mathew et al. in 2020 showed that SBRT exerted long-lasting tumor-control effects on patients at different stages, with the OS of 31.7 and 23.2 months for patients with Child–Pugh classes A and B/C, respectively; the study analyzed the survival data of 297 North American patients with HCC without macrovascular invasion^26^. Conversely, in a study by Hong et al. patients with HCC who underwent RT showed a median OS of 19.5 months and a 2-year local control rate of 94.8%^27^. Owing to studies on Asian populations, RT is a promising strategy for improving median survival time. A phase 2 clinical study on the efficacy of SBRT in patients with unresectable HCC from Japan showed that the patients receiving SBRT had a median OS of 41.7 months (range: 6.8–96.2 months) and a 3-year local control rate of 96.3%^28^. These findings are consistent with our findings, suggesting that RT offers patients an opportunity to survive longer in a diverse population of patients with inoperable HCC.

Here, we considered age as an independent predictor of OS in patients with HCC in baseline demographics, with younger patients having longer OS, which was consistent with previous findings^29,30^. It is evident that younger patients (< 70 years old) who underwent RT had the longest survival time, and RT also markedly improved survival in older patients. Generally, younger patients have better liver regeneration capacity, and aging reduces liver mass, blood flow, and the number of hepatocytes, thus leading to a much higher risk of radiation-induced liver diseases^31,32^. Our results suggest that RT remains one of the viable options for older patients with HCC exhibiting adequate liver functions.

Accumulating evidence suggests that most patients with HCC can be treated with different RTs, such as SBRT, IMRT, or GKR, regardless of the tumor location^33,34^; however, existing guidelines still recommend RT only for patients with no indications for surgery or ablation in the early to mid-stage or who do not want to undergo invasive treatment. Therefore, we selected patients with HCC without distant metastases as our study population, and consistent with our expectations, RT significantly prolonged survival in this group of patients, especially in those with early-stage and more differentiated tumors. These findings are also consistent with the results of previous studies^35,36^. Moreover, the majority of patients treated at our hospitals underwent GKR as their RT modality. GKR, an external RT method, is the most prevalent form of stereotactic radiosurgery in America, attributed to its precise and efficient delivery of radiation in a single session. However, its adoption in China remains constrained^19^. Our study shows the efficacy of GKR in patients with inoperable HCC, potentially facilitating its application to inoperable HCC cases in China, and providing valuable insights into informed clinical decision-making.

The present study has some shortcomings and limitations. First, part of the data was obtained from the SEER database. SEER-based analysis has a large sample size; however, it lacks detailed clinical information; for example, whether or not the patient underwent chemotherapy, because the SEER database does not include information on chemotherapy regimens. Second, both the patient data from the SEER database and our hospitals were analyzed retrospectively in our study; thus, there may be more potential confounding factors. Despite the PSM analysis, the effect of such factors could not be completely excluded. Third, the number of patients from our hospitals (sample size) was relatively small, which might lead to some deviations in result interpretation.

## Conclusion

In this multicenter retrospective study, we used the SEER database and patient data from three hospitals in China and showed that RT leads to improved survival outcomes in patients with inoperable HCC. Hence, a multidisciplinary approach that encompasses RT should be considered while managing patients with HCC. To further confirm the safety and generalizability of RT, additional prospective randomized controlled clinical trials need to be conducted.

## Supplementary Information

Supplementary Figures.

## Data Availability

The datasets generated or analyzed during the study are available from the corresponding author on reasonable request.
