# Supplementary material for: A propensity score matching study on survival benefits of radiotherapy in patients with inoperable hepatocellular carcinoma
Source: Sci Rep. 2023 Apr 27;13:6879. doi: 10.1038/s41598-023-34135-6 (PMC10140032; doi:10.1038/s41598-023-34135-6)
Supplement: Supplementary file 1 — Supplementary Figures. [file 41598_2023_34135_MOESM1_ESM.docx]

**Supplementary Material**

A propensity score matching study on survival benefits of radiotherapy in patients with inoperable hepatocellular carcinoma

Hao Zeng, Ke Su, Xiaojing Chen, Xueting Li, Lianbin Wen, Yanqiong Song, Lan Chen, Han Li, Lu Guo, Yunwei Han


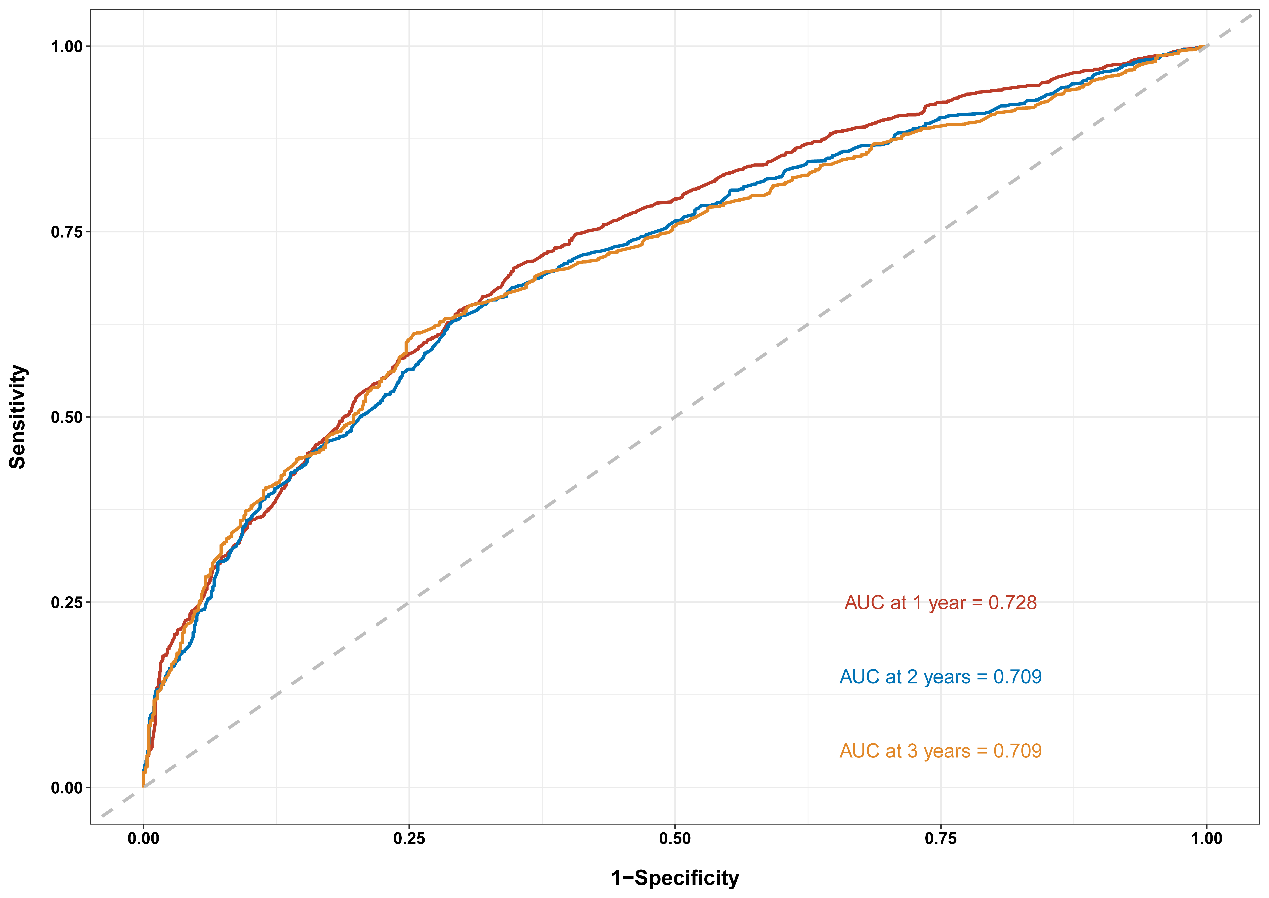


**Fig S1.** Time-dependent receiver operating characteristic curves for validation set.


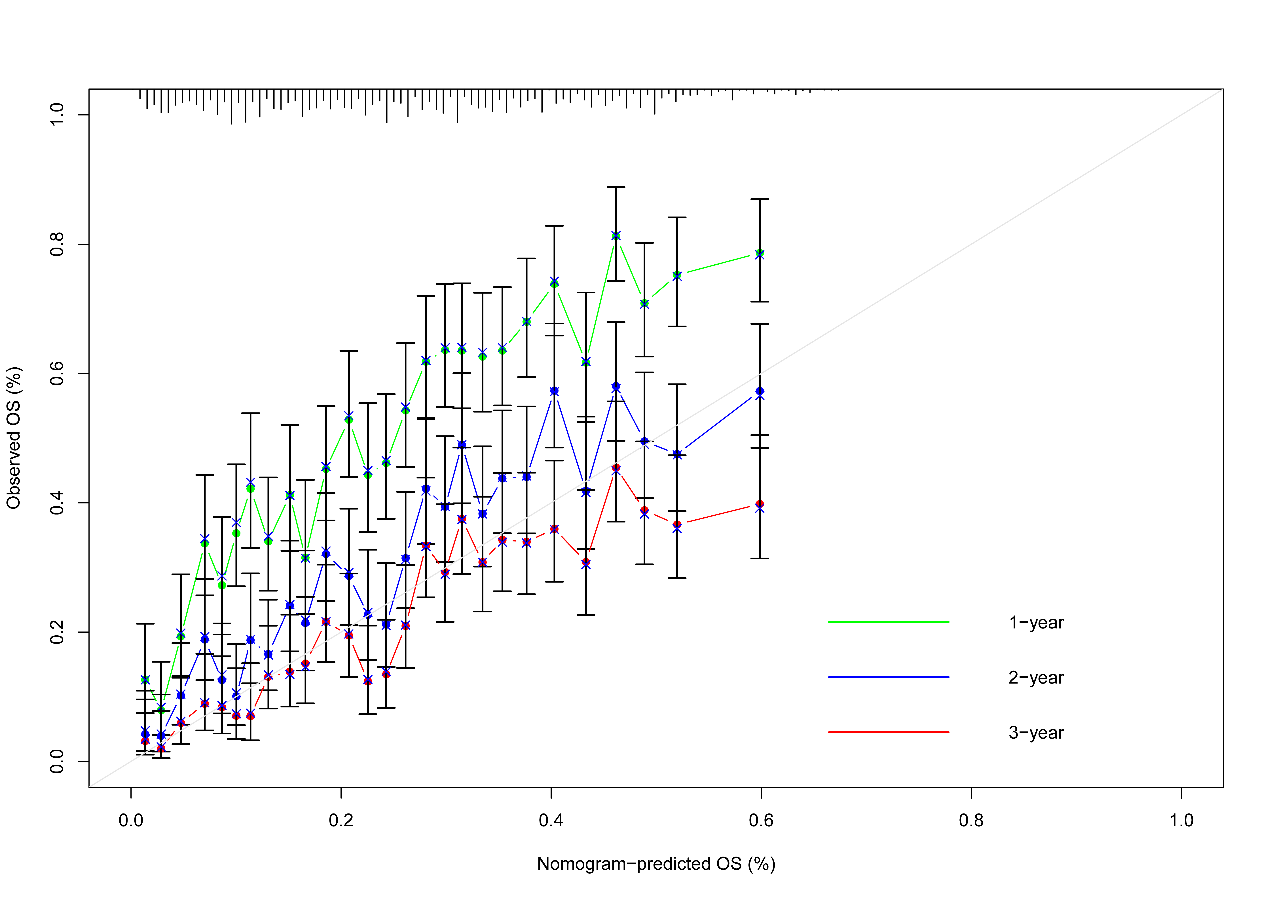


**Fig S2.** Calibration curves in the validation cohorts.

**
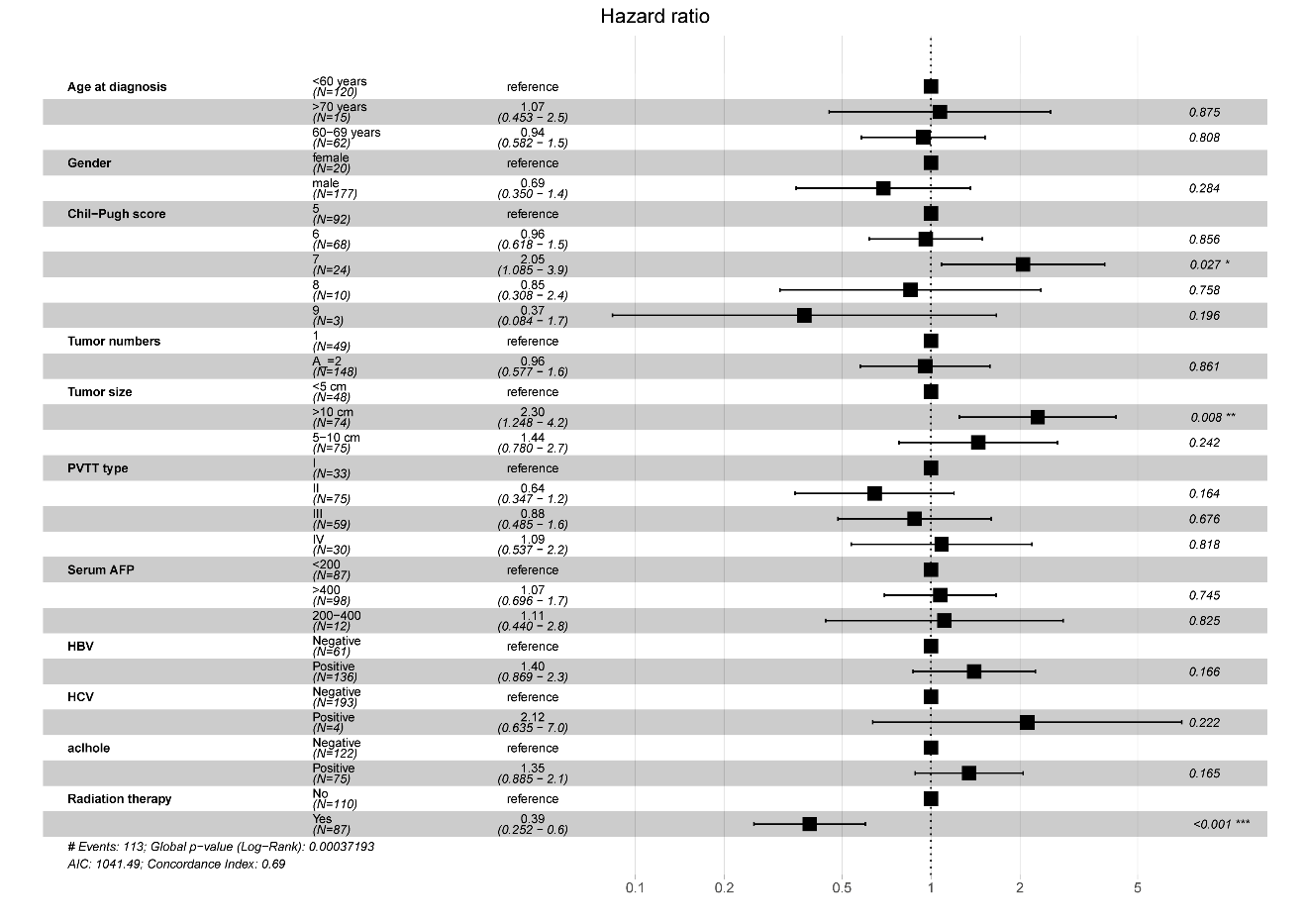
Fig S3.** Cox proportional hazard ratios with 95% confidence intervals for data from patients at three tertiary hospitals in China.


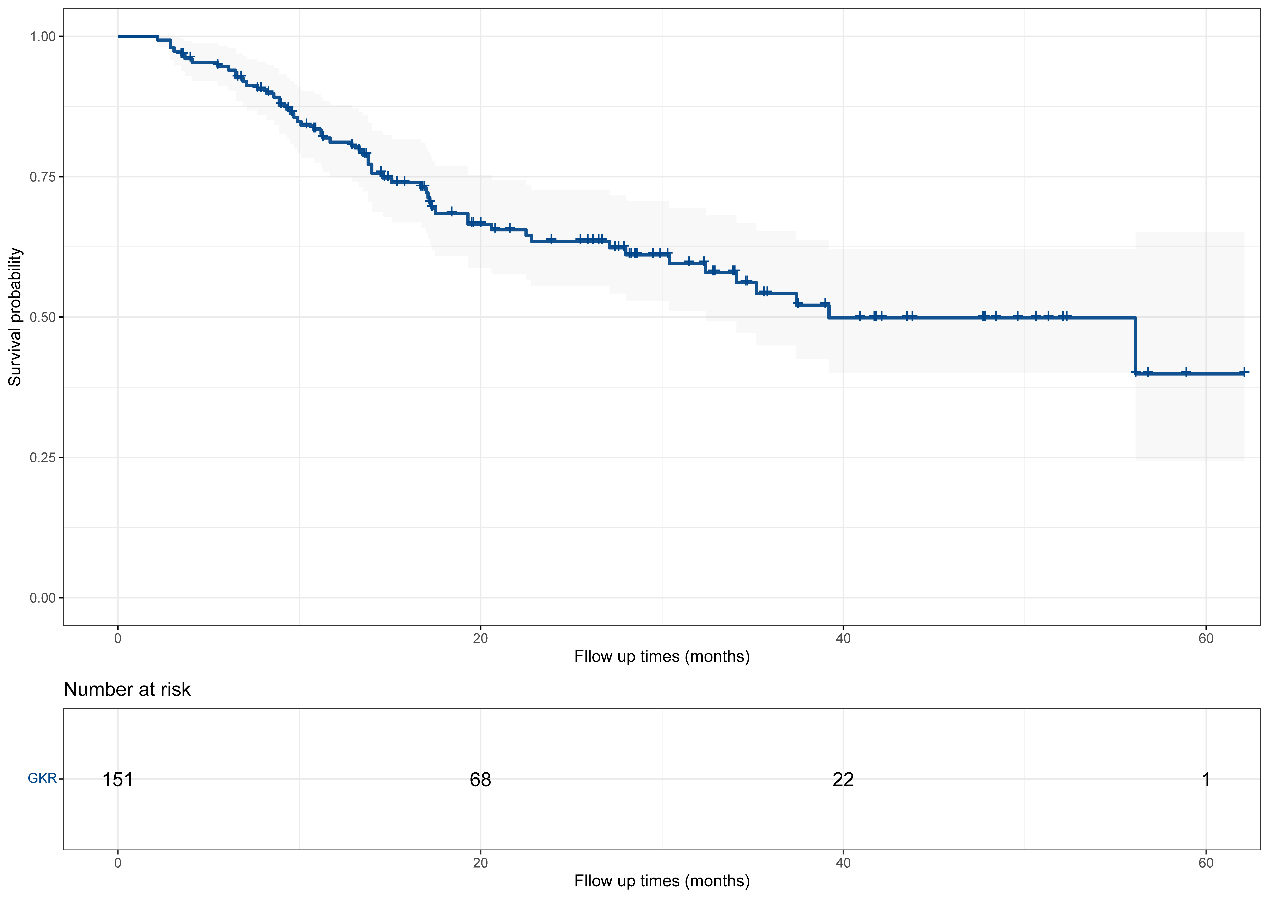


**Fig S4.** Kaplan-Meier overall survival (OS) estimates and 95% confidence intervals for patients in GKR group at three tertiary hospitals in China. GKR, gamma knife radiosurgery.
